# Binding Behavior between Transforming-Growth-Factor-Beta1 and Its Receptor Reconstituted in Biomimetic Membranes

**DOI:** 10.3390/membranes13040446

**Published:** 2023-04-19

**Authors:** Gounhanul Shin, Kunn Hadinoto, Sungmun Lee, Jin-Won Park

**Affiliations:** 1 Department of Chemical and Biomolecular Engineering, College of Energy and Biotechnology, Seoul National University of Science and Technology, Seoul 01811, Republic of Korea; 2 School of Chemical and Biomedical Engineering, Nanyang Technological University, Singapore 639798, Singapore; 3 Department of Biomedical Engineering, Khalifa University of Science and Technology, Abu Dhabi P.O. Box 127788, United Arab Emirates

**Keywords:** TGF-β1, biomimetic membranes, binding

## Abstract

Transforming growth factor β1 (TGF-β1) is critical to cell differentiation, proliferation, and apoptosis. It is important to understand the binding affinity between TGF-β1 and its receptors. In this study, their binding force was measured using an atomic force microscope. Significant adhesion was induced by the interaction between the TGF-β1 immobilized on the tip and its receptor reconstituted in the bilayer. Rupture and adhesive failure occurred at a specific force around 0.4~0.5 nN. The relationship of the force to loading rate was used to estimate the displacement where the rupture occurred. The binding was also monitored in real time with surface plasmon resonance (SPR) and interpreted with kinetics to acquire the rate constant. Using the Langmuir adsorption, the SPR data were analyzed to estimate equilibrium and association constants to be approximately 10^7^ M^−1^ and 10^6^ M^−1^ s^−1^. These results indicated that the natural release of the binding seldom occurred. Furthermore, the degree of binding dissociation, confirmed by the rupture interpretation, supported that the reverse of the binding hardly happened.

## 1. Introduction

Transforming-growth-factor-beta1 (TGF-β1) is a cytokine that plays multifunctional roles in major regulatory signaling pathways [1]. Once TGF-β1 ligands bind to the extracellular domains of their receptors, the receptors form a serine/threonine kinase complex with other factors [2]. This complex activates a signaling cascade for biological processes such as development, proliferation, differentiation, and migration [3]. Since the pathway is involved in these processes, its inhibition has been considered as a potential drug target [4]. Two theoretical approaches—kinase targeting and formation interruption—have potential for this inhibition. Most of the current approaches are based on kinase targeting, which lacks specificity and can lead to side effects. Therefore, formation interruption via the disruption of the binding of the TGF-β1 ligands to their receptors was investigated. However, the mechanism behind the activation is little understood.

Supported lipid bilayers have been widely utilized as biomimetic platforms, where membrane protein has been reconstituted for further investigations on apoptosis, transport, signal transduction, and energy production [5,6,7,8]. These bilayers are accessible to analytical techniques such as cyclic voltammetry, fluorescence microscopy, fluorescence recovery after photobleaching, infrared reflection absorption spectroscopy, neutron reflectometry, quartz crystal micro-balance, surface-enhanced infrared absorption spectroscopy, atomic force microscopy (AFM), and surface plasmon resonance (SPR) [9,10,11,12,13,14,15,16,17]. AFM has been used to investigate the morphology and the mechanics of these bilayers with the surface force measurements under physiological conditions, while SPR allows for the real-time monitoring of their formation and interaction in a label-free manner and with pM-scale sensitivity. In this study, we wanted to investigate the binding information between TGF-β1 and its receptor reconstituted in biomimetic membranes. This information—force, kinetics, and equilibrium—may represent a novel approach for diagnosis or therapy associated with TGF-β1.

## 2. Materials and Methods

TGF-β1 receptor Ⅱ (Cat. No, HY-P78524, MedChemExpress) was reconstituted in biomimetic membranes made with 1,2-dipalmitoyl-*sn*-glycero-3-phosphothioethanol (DPPTE) and dioleoylphosphatidylcho line (DOPC) (Sigma Aldrich, St. Louis, MO, USA). First, a lipid monolayer was formed via thiol-tethering between lipid headgroups and gold surfaces. Then, another lipid monolayer was created on the formed previously monolayer. This creation was generated through vesicle fusion via hydrophobic interaction. Last, the binding site to TGF-β1 was only distributed on the outer layer. Gold surfaces (BIAcore SA, Little Chalfont, Buckinghamshire, UK) were covered with a solution (sulfuric acid:hydrogen peroxide = 3:1) at 60 °C for 10 min and dried with nitrogen stream immediately before the surfaces were immersed in 2 mL of chloroform of 1 mM DPPTE and 0.5 mM DOPC for 2 h. After DOPC vesicles were prepared for fusion into a 10 mg/mL lipid concentration and analyzed according to the procedures published previously, 10 μL of 1 μg/mL TGF-β1 receptor Ⅱ in ethanol was added to 1 mL of the vesicle solution for reconstitution [18].

Force measurements were performed identically to the procedures published previously [19]. Prior to the TGF-β1 immobilization, the cantilever was treated with 0.1 mM 16-mercaptohexadecanoic acid and 0.9 mM 1-mercapto-1-undecanol (MUD) in ethanol and an ethanoic solution of 15 mg/mL 1-ethyl-3-(3-dimethylaminopropyl)-carbodiimide and 6 mg/mL *N*-hydroxysuccinimide in sequence. Then, the cantilever was rinsed with DI water and incubated with 0.1 mg/mL TGF-β1 for 2 h. The forces were measured between the cantilever and the reconstituted membrane and collected as curves with respect to a cantilever position. Only the curves with the highest adhesion among the collections were interpreted. SPR was observed following the procedures described in the previous publication [20]. The membrane formation was repeated with identical steps to the force measurement above. The concentrations of TGF-β1 were 0.05, 0.1, 0.2, 0.5, 1.0, 2.0, and 5.0 μM.

## 3. Results

### 3.1. Direct Adhesion

**Figure 1:**
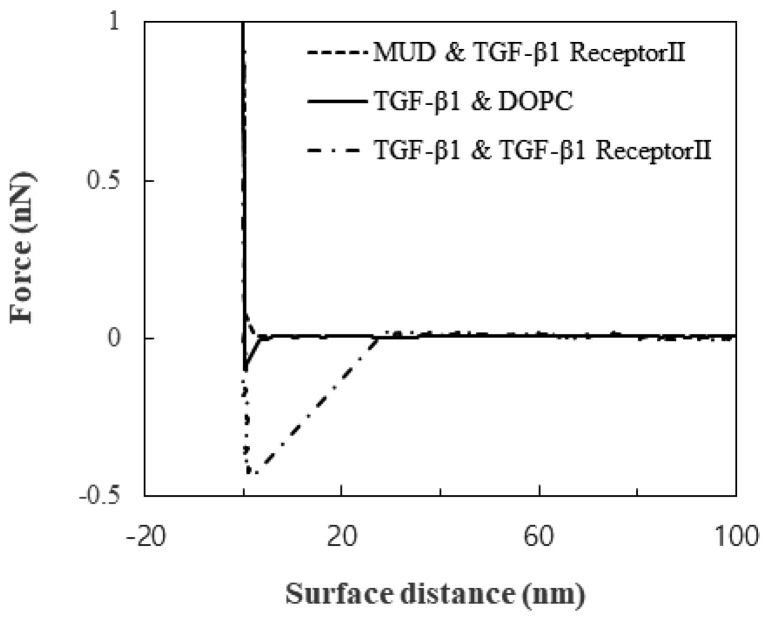
Force as a function of the surface distance.

Little substantial attraction was observed between TGF-β1 receptor Ⅱ and the MUDs, and this observation showed that the MUDs made a great background for measuring the TGF-β1-specific interaction. In contrast to the attraction between TGF-β1 receptor Ⅱ and the MUDs, the attraction between the DOPC layer and the TGF-β1 immobilized on the tip was 0.06 to 0.1 nN. The attraction between TGF-β1 and TGF-β1 receptor Ⅱ was clearly distinguished: 0.4 to 0.5 nN. These attractions are shown in the plot of the force versus the surface distance (Figure 1). Not only the magnitude of the attraction but also the snap-off was significantly different. Therefore, the attraction of the DOPC layer seemed to be non-specific membrane-adhesion force, while the attraction of TGF-β1 receptor Ⅱ did seem to be specific.

The strong snap-off may have arisen from either the rupture of the specific TGF-β1 bond or the detachment of TGF-β1 receptor Ⅱ from the membrane. Since the attraction was measured repeatedly, the surface was reversibly modified by the measurements. Therefore, the snap-off resulted from the rupture. Furthermore, previous studies suggested that the lipid layer on the solid substrate was unperturbed with loads of more than 0.6 nN [21]. Therefore, the specific bond between TGF-β1 and its receptor was ruptured before the lipid bilayer was disturbed. The range of 0.4 to 0.5 nN was attributed to the change in the orientations of TGF-β1 and its receptor and their location during the load.

### 3.2. Real-Time Binding

**Figure 2:**
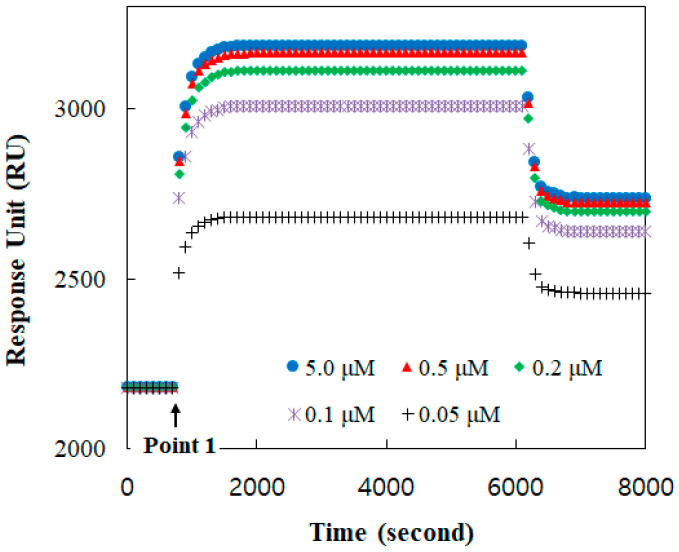
Binding responses in real time between TGF-ß1 and TGF-ß1 receptor Ⅱ reconstituted in the DOPC bilayer for TGF-ß concentrations of 0.05, 0.1, 0.2, 0.5, 1.0, 2.0, and 5.0 μM.

The binding formation between TGF-β1 and its receptor was monitored with respect to time using the SPR response, as shown in Figure 2. A baseline, before point 1, was established at the receptor reconstituted under the buffer solution. At point 1, the TGF-β1 solution was injected. The amount of the bound TGF-β1 was increased with its concentration. The TGF-β1 receptors were mostly occupied at a solution of more than 0.5 μM TGF-β1. Therefore, the response at 2 μM was clearly presumed to be for all receptors.

## 4. Discussion

**Figure 3:**
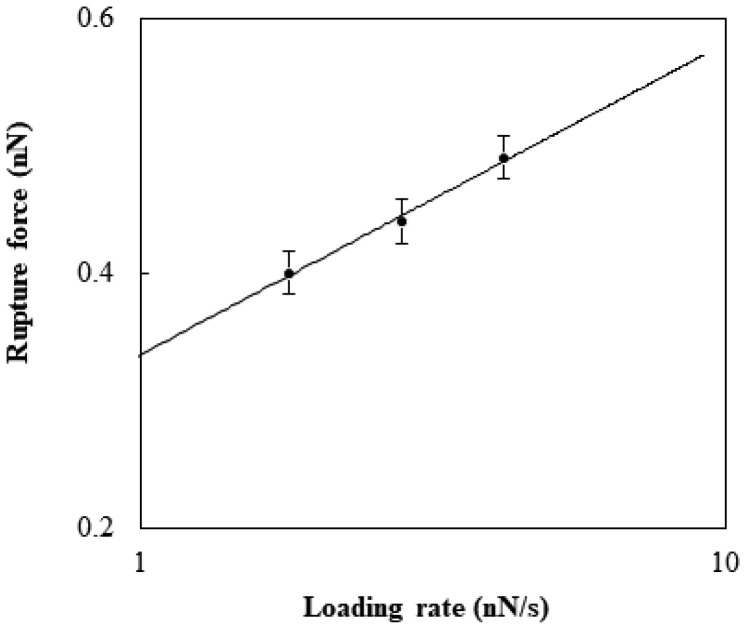
Rupture force of the specific binding between TGF-β1 and TGF-β1 receptor Ⅱ reconstituted in the DOPC bilayer with respect to the logarithm of the loading rate.

The rupture was affected by the loading rate. As the rates, 1.8, 2.8, and 4.2 nN/s were selected and estimated by multiplying the retraction rate by the cantilever spring constant. The force to generate the rupture at a given loading rate was considered with the theoretical model developed by Kramer [22]. The rupture force, *F* (N), has the following relation with the loading rate, *r* (N/s):

(1) $$F=\frac{k_{B}T}{x}\ln\left(\frac{rx}{k_{B}T\cdot k_{off}}\right)$$

where *k_B_* is the Boltzmann constant (J/K), *T* is the temperature (K), *x* is the potential width between the bound state and the transition state (m), and *k_off_* is the natural dissociation rate at zero force (1/s). The fitting of Equation (1) was performed to estimate the values of *k_off_* and *x* for the specific binding between TGF-β1 and its receptor (Figure 3). The rupture displacement was about 0.1 to 0.13 nm, which was in close agreement with the value found previously [23]. The natural dissociation rate was about 0.3 to 0.4 s^−1^. Previous research performed with AFM suggested that the interaction was around 0.1~0.16 nN and the natural dissociation rate was 0.66 s^−1^ [24]. This difference seemed to be caused by the cooperation of the extracellular domain with the receptors, because the previous research was on cell surfaces.

For the kinetic interpretation of the SPR binding, the coverage fraction from the response was considered with respect to time [17].

(2) $$\ln\left(1-c\right)=-kt$$

where *c* is the coverage fraction, *k* is the rate constant (1/s), and *t* is the time (s). Equation (2) was fitted to the results of ln (1 − *c*) with respect to time. From this fitting, the rate constant was 6.5∼7.5 × 10^−3^ s^−1^ with a determination coefficient of 0.986. The rate constant could have shown how many active receptors were located on the biological membrane with a given concentration of TGF-β1.

**Figure 4:**
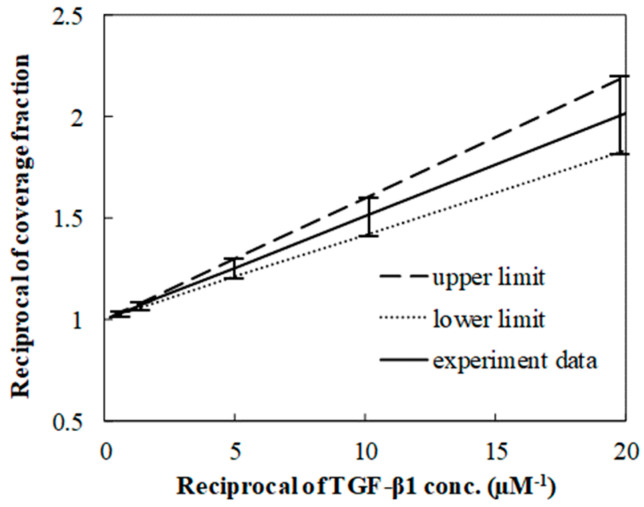
Linear relation between reciprocal of coverage fraction and reciprocal concentration. The linear relation is located in the range of the limits.

Langmuir isotherms were used to estimate the equilibrium constant of the TGF-β1 SPR binding to its receptor, which was from fitting the fraction with respect to the TGF-β1 concentration. Since the linear fit was essential to estimate the constant, the fraction and the concentration became the reciprocal form (Figure 4 on the next page). The constant was in the range of 1 × 10^7^ to 3 × 10^7^ M^−1^, which was close to the value of around 10^7^ M^−1^ which was found in the results of the previous research [25,26]. Both lower and upper limits were extracted from the extreme values of the ranges where the data were distributed.

Because *k_off_* was found from the analysis of the rupture force, the association rate was estimated to be approximately 10^6^ M^−1^ s^−1^. This rate was also close to the results published previously [25]. The association rate seemed consistent with the rapid increase in the SPR curve right after the TGF-β1 injection, while a slight decrease was observed for more than 1000 s after the equilibrium was reached. This interpretation suggested that little reverse reaction happened.

Since the TGF-β1 coverages were acquired under the condition of the unbound TGF-β1 and its receptor, the reverse reaction may be still of concern. However, the analysis of the rupture force indicated little occurrence of the reaction. The binding energy, estimated from the multiplication of the rupture force and the displacement, was approximately 5.0 × 10^−19^ J, 200 times higher than the thermal fluctuation at room temperature. TGF-β1 may have only been released from its receptor if the binding received more than 200 times the energy, unless any denaturation occurred.

## 5. Conclusions

In this study, TGF-β1 formed specific binding with its receptor. The binding force between TGF-β1 and its receptor was measured using AFM. The significant adhesion, around 0.4~0.5 nN, was induced by the interaction between the TGF-β1 immobilized on the tip and its receptor reconstituted in the bilayer. The relation between the rupture force and the loading rate showed that the rupture displacement was about 0.1 to 0.13 nm, which was in the range of a van der Waals bond. The binding was also monitored in real time with surface plasmon resonance (SPR) and interpreted with kinetics to acquire the rate constant. The analysis of the SPR data with the Langmuir adsorption showed that the equilibrium and association constants were approximately 10^7^ M^−1^ and 10^6^ M^−1^ s^−1^. These results indicated that the natural release of the binding seldom occurred. Furthermore, the degree of binding dissociation, confirmed by the rupture interpretation, supported the concept that the reversal of the binding hardly happened. The information found in this research may be useful to investigate TGF-β1-mediated biological processes. For example, the overexpression of TGF-β1 has been found to cause some diseases related to the kidneys. If the binding of TGF-β1 is quantitatively determined, its analog function could be precisely considered.

## Data Availability

Not applicable.
